# Silyl-protective groups influencing the reactivity and selectivity in glycosylations

**DOI:** 10.3762/bjoc.13.12

**Published:** 2017-01-16

**Authors:** Mikael Bols, Christian Marcus Pedersen

**Affiliations:** 1 Department of Chemistry, University of Copenhagen, Universitetsparken 5, 2100 Copenhagen, Denmark

**Keywords:** Carbohydrate, conformation, glycosylation, reactivity, selectivity

## Abstract

Silyl groups such as TBDPS, TBDMS, TIPS or TMS are well-known and widely used alcohol protective groups in organic chemistry. Cyclic silylene protective groups are also becoming increasingly popular. In carbohydrate chemistry silyl protective groups have frequently been used primarily as an orthogonal protective group to the more commonly used acyl and benzyl protective groups. However, silyl protective groups have significantly different electronic and steric requirements than acyl and alkyl protective groups, which particularly becomes important when two or more neighboring alcohols are silyl protected. Within the last decade polysilylated glycosyl donors have been found to have unusual properties such as high (or low) reactivity or high stereoselectivity. This mini review will summarize these findings.

## Introduction

**Figure 1:**
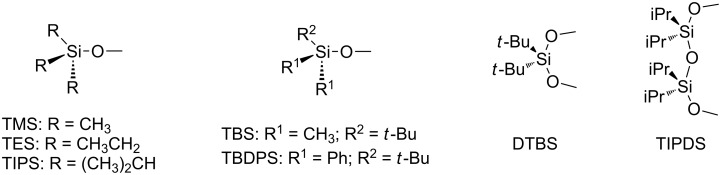
Silicon-protective groups typically used in carbohydrate chemistry.

Silicon-based protective groups of alcohols have a long history in organic chemistry [1–3]. The most popular and commercially available silyl-protective groups are trimethylsilyl (TMS), triethylsilyl (TES), *tert*-butyldimethylsilyl (TBS), *tert*-butyldiphenylsilyl (TBDPS), triisopropylsilyl (TIPS) as well as the diol-protective groups DTBS and TIPDS (Figure 1). Silyl groups have also early been used in the carbohydrate field to provide an alternative orthogonal protective group to the more conventional acetyl, benzoyl and benzyl groups. Particularly in oligosaccharide synthesis where many orthogonal hydroxy protective groups are required silicon protective groups have frequently been introduced in both glycosyl donors and acceptors. However, glycosylation with heavily silylated carbohydrate derivatives is comparatively new, and so is the significance that silyl groups have on the stereoselectivity and reactivity in glycosylation reactions [4]. These findings, which most have occurred in the last decade, will be reviewed here.

## Review

**Scheme 1:**
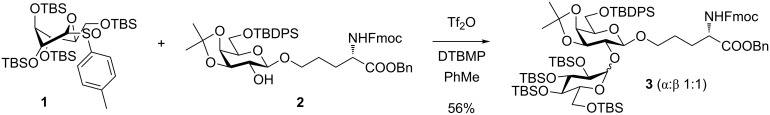
Glycosylation with sulfoxide **1**.

One of the earliest glycosylations with a persilylated glycosyl donor was carried out by Kihlberg and Broddefalk who needed an acid-labile protective group [5]. They protected a thiocresyl glucoside with TBS groups, oxidized the sulfur to sulfoxide **1** and used the latter to glucosylate the 2-OH of the galactose derivative **2** (Scheme 1). The reaction gave a 56% yield of **3** as a 1:1 mixture of α- and β-glucosides. Migration of a TBS group to the acceptor alcohol **2** was observed as a byproduct (10%). Attempts of glycosylating **2** with the thioglycoside or the corresponding glycosyl halides were unsuccessful. NMR studies of **1** revealed that the compound adopted a skew-boat conformation, based on the small ^3^*J* coupling constants, as well as long range w-couplings. This conformational flip is induced by the presence of the bulky *trans*-vicinal silyl groups [6].

**Scheme 2:**
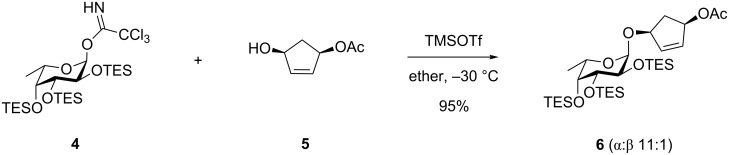
Glycosylation with imidate **4**.

Also with the purpose of having acid-labile protective groups on the donor a TES-protected trichloroacetimidate of fucose, **4**, was employed by Myers et al. [7] in order to have protective groups compatible with their synthesis of neocarzinostatin. It was found that optimal glycosylation was performed with TMSOTf as a catalyst at low temperature and excess donor in diethyl ether since this gave the best α-selectivity (Scheme 2). Using other protective groups on the fucose part, such as 2,3-TIPDS and 4-*O*-TES led to glycosylation with only poor stereoselectivity [8]. The TES groups were also used successfully on the 2-methylamino analogue of **4**.

**Scheme 3:**
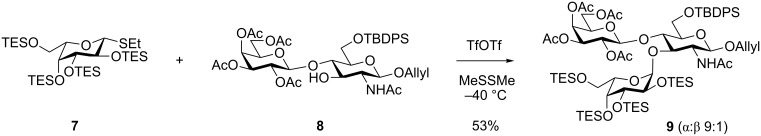
Glycosylation with thioglycoside **7**.

A glycosylation with a TES-protected glycosyl donor has also been performed in a case where the target contained a 6-*O*-acylglucoside and hence protective groups that could be removed under mild acidic conditions were needed [9]. This was for example used for the synthesis of the serine protease inhibitor banyaside. TES-protected glycosylimidates were also employed in the synthesis of antitumor saponins which contained partially acylated oligosaccharides. The TES groups could be removed by comparatively mild treatment with fluoride without hydrolysis or migration of *O*-acyl groups [10]. This strategy has also been applied to prepare partially acylated cholestan glycosides. In this case an imidate with a 2-*O*-acetate and 3,4-*O*-TES protection was used, which ensured stereoselectivity by neighboring-group participation [11]. For similar reasons the per-TES-protected thioglycoside **7** was employed to prepare the Lewis X trisaccharide: The reaction of **7** with disaccharide **8** promoted by dimethyl disulfide and triflic anhydride gave trisaccharide **9** with high α-selectivity (Scheme 3) [12]. These conditions, using this promoter system, worked fine in a number of similar cases.

**Scheme 4:**
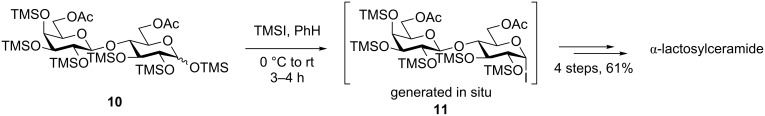
In situ formation of a silylated lactosyl iodide for the synthesis of α-lactosylceramide.

The less-stable trimethylsilyl group has been employed by Gervay–Hague and co-workers to protect glycosyl donors [13–17]. The reaction of a hexa-TMS-protected lactose derivative **10** with TMS iodide converted it to glycosyl iodide **11** that glycosylated alcohols in good yields (Scheme 4). The TMS protective groups are however rather unstable and they were exchanged to acetyl groups after the glycosylation step [13]. Nevertheless, the TMS-protected glycosyl iodides were useful intermediates because they were more reactive and less prone to elimination than the corresponding benzylated or acetylated glycosyl iodides.

### Effect of silyl protective groups on the reactivity

**Figure 2:**
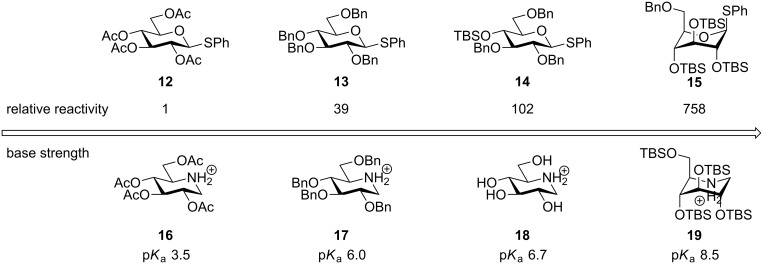
Comparison of the reactivity of glycosyl donors with the p*K*_a_ of the corresponding piperidinium ions.

Protective groups can profoundly influence the reactivity of carbohydrate derivatives and especially glycosyl donors [18]. This influence is due to the different electron-withdrawing capability of protective groups. During the glycosylation reaction the anomeric carbon becomes increasingly electron poor, with the formation of a glycosyl cation as the extreme. This development of a (partial) positive charge is less favorable with more EWD protective groups and the reaction becomes slower; i.e., the donor is less reactive (disarmed) [19]. Ester protective groups such as acetyl and benzoyl are among the most electron-withdrawing of the common protective groups, whereas benzyl (or methyl) groups are less so, which is reflected in the reactivity of glycosyl donors carrying these groups. As shown in Figure 2, the thioglycoside with benzyl ethers **13** is about 40 times more reactive towards glycosylation with methanol upon activation by NIS, than the acetylated counterpart **12**, but the thioglycosides with silyl ethers are even more reactive [20].

**Figure 3:**
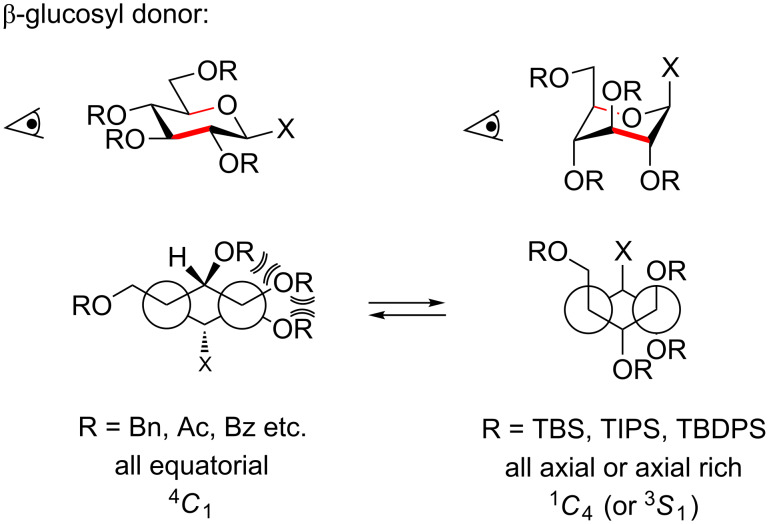
Conformational change induced by bulky vicinal protective groups such as TBS, TIPS and TBDPS. The vicinal clash overrules the 1,3-diaxial interaction, which is less influenced by bulky silyl ethers as these can rotate more freely in the axial-rich conformation. The projections are along the red bonds in the two models.

Thus the presence of a single *O*-TBS group (**14**) can more than double the reactivity while three (**15**) will increase the rate by 20 times as compared to benzyl. The increased reactivity of the silylated glycosyl donors is partially due to the *O*-silyl group being somewhat less electron withdrawing than the benzyl, but also due to the ability of bulky silyl groups to cause a change in the sugar ring conformation [21]. The influences of the various protective groups are also clearly reflected in their ability to alter the base strength of the transition state mimicking amine deoxynojirimycin (Figure 2) [22]. The acetylated amine **16** is vastly less basic than the benzylated analogue **17**, which is still less basic than the unprotected amine **18** which in many ways should be similar to an O-silylated compound **19** since the silyl group inductively is very comparable to the proton. Yet the silylated amine **19** is almost a 100 times more basic due to the conformational ring flipping induced by the bulky silyl groups. This extraordinary effect on the basicity and the donor reactivity stems from the conformational change in the sugar ring, which causes the OR groups in the 3 and 4 and occasionally the 2-position to adopt an (pseudo)axial orientation, which is less electron withdrawing [23]. This conformational change is induced when having *trans*-vicinal OR groups (Figure 3). Normally the bisequatorial orientation is preferable due to 1,3-diaxial interactions of axial substituents. This steric interaction can however be overridden when the R groups are sufficient bulky and hence the sugar ring changes the conformation. The electronegativity of the R group is probably also important; when more electropositive (as Si), the oxygen atoms become more electron rich and their repulsion becomes larger.

Changing the conformation of a heterocycle has, as mentioned, been studied using the piperidine model system. The p*K*_a_ of the corresponding piperidinium ion is a measure of the stereoelectronic effects and correlates with the glycosyl donor’s reactivity observed. Forcing an OR group from an equatorial position into an axial position by, e.g., a bulky silyl group, increases the basicity of the piperidines, which is analogous to increasing the reactivity of the corresponding glycosyl donors.

The increased reactivity is very clearly displayed when TBS or TIPS-protected thioglycosyl donors are mixed with benzylated thioglycoside acceptors under activating conditions (Table 1). The benzylated thioglycosides **21** and **26**, normally termed ‘armed’ due to their comparatively high reactivity, were selectively glycosylated by silylated thioglycosides (**20**, **23**, **25**, **28** and **30**) in high yield without any self-glycosylation of the armed donors [24–25]. Based on their extraordinary reactivity these silylated donors were termed ‘superarmed’. The listed reactions (Table 1) were all highly stereoselective as well. The stereoselectivity is very dependent on the bulkiness of the protective group on C2 in the mannosyl (**28**), rhamnosyl (**23** and **30**) and glucosyl donors (**20**) (see also Scheme 11). In these systems the *trans* products are favored. In the galactosyl donor **25** the bulky C4 substituent shields the β-face of the donor and hence the glycosylation is very α-selective.

**Table 1:** Reaction of silylated thioglycosides with benzylated thioglycoside acceptors.

Silylated donor	Benzylated donor	Product^a^ (yield %)

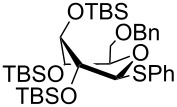 **20**	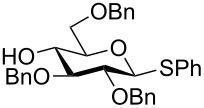 **21**	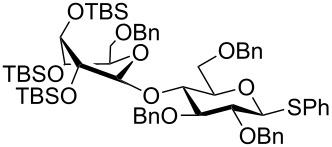 **22** (85%)
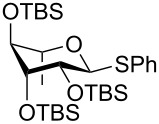 **23**	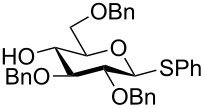 **21**	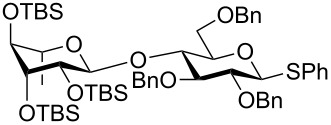 **24** (90%)
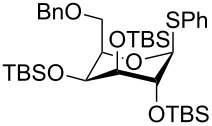 **25**	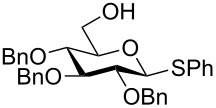 **26**	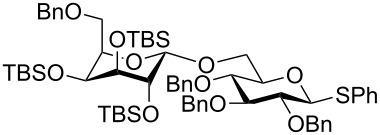 **27** (70%)
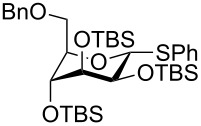 **28**	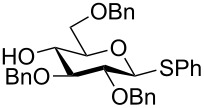 **21**	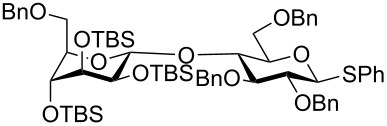 **29** (81%)
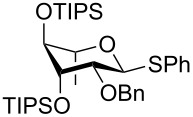 **30**	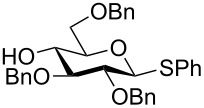 **21**	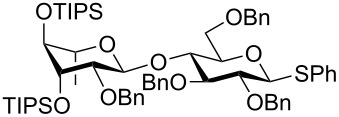 **31** (66%)

^a^Only the shown stereoisomer was obtained. Data taken from [24–25].

**Scheme 5:**
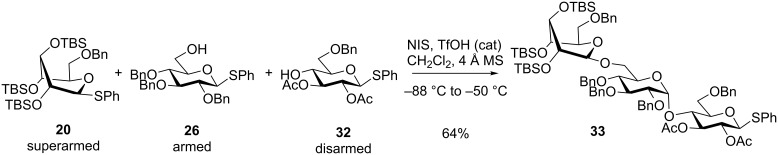
An example of a “one pot one addition” glycosylation, where 3 glucosyl donors are mixed with 2.1 equiv NIS and a catalytic amount of TfOH. The individual donors are activated at different temperatures due to their reactivity and the trisaccharide donor is formed in an excellent yield.

The remarkable difference in reactivity between disarmed, armed and superarmed donors **20**, **26** and **32**, respectively was used for “one-pot one addition” glycosylations having all 3 donors present together with all reagents from the start (Scheme 5). The activation of the individual donors was controlled by changing the temperature and the trisaccharide donor **33** could thereby be prepared in excellent yields [21].

The reactivity of silylated donors have also been investigated by Hung, Wong and collaborators [26]. Investigating benzylated thioglucosides with a single or two TBS or TIPS groups in different positions they observed an increasing rate that were qualitatively similar to those described in Figure 2. Rate increases were however larger and TIPS protection had a greater rate-increasing effect than TBS protection.

The rate increases caused by a single silyl group in the 2,3 and 4-position are particular remarkable given that no obvious conformational change in the ground state is observed. Thus the increased rate must be caused by the group’s ability to favor conformational inversion to the more reactive axial conformation in the transition state. This explains the comparatively large rate enhancements observed by TBS and TIPS groups compared to unprotected OH and also that TIPS, which is more bulky than TBS, but essentially has the same inductive effect, causes a greater rate enhancement.

Gervay–Hague has reported that TMS-protected glycosyl iodides are remarkably more reactive than their benzyl-protected analogues [13,27]. While this rate enhancement is at least partially stemming from the change in the inductive effect, it is also possible that the comparatively more bulky TMS groups also cause an enhancing effect by favoring conformational inversion to the stereoelectronically more stable conformer in the transition state.

**Scheme 6:**
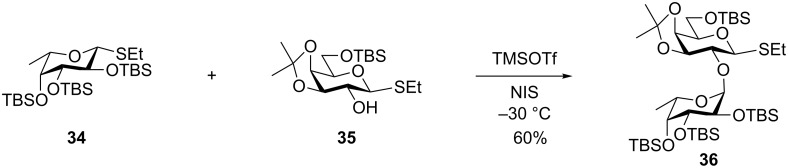
Superarmed-armed glycosylation with thioglycoside **34**.

The reactivity of TBS-protected thioglycosides was further investigated by Scanlan and co-workers who made the fucosyl donor **34** (Scheme 6) [28]. Interestingly the NMR spectrum of this compound displayed line broadening indicating some conformational inversion, but the X-ray structure of the crystalline compound was in the conventional ^1^*C*_4_ conformation. Yet the compound was clearly very reactive as it selectively could glycosylate the 2-OH of thioglycoside **35** giving **36** in a very good yield. Other acceptor alcohols were also glycosylated in a good yield and with high α-selectivity [28].

**Scheme 7:**
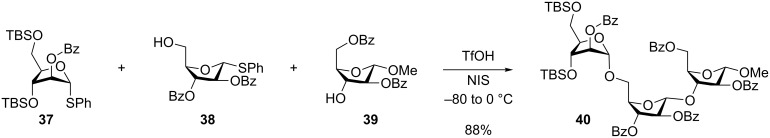
One-pot double glycosylation with the conformationally armed thioglycoside **37**.

Yang and co-workers have extended the concept to the furanoside series [29]. They showed that the arabinofuranosyl donor **37** and its 2-*O*-TBS analogue were more reactive than the corresponding benzylated thioglycosides in competition reactions and used the reactivity differences in a one-pot glycosylation reaction between **37**, a disarmed donor/acceptor **38** and an acceptor **39**, which gave the trisaccharide **40** in a remarkable yield of 88% (Scheme 7). This reaction works so well because the more readily activated donor **37** reacts with the more reactive and accessible primary alcohol of **38** rather than with the secondary hydroxy group in **39**.

**Scheme 8:**
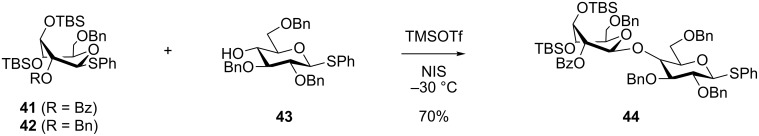
Superarmed-armed glycosylation with thioglycoside **41**.

In the above study it was found that **37** was less reactive than the persilylated analogue [29], which was not obvious as the Demchenko group [30–35] has shown that a 2-*O*-ester can have an activating effect by the aid of anchimeric assistance [36]. This combination of conformational arming and anchimeric assistance was investigated by Heuckendorff et al., who studied the 2-*O*-benzoylated analogue of **20**, **41** (Scheme 8) [37]. They observed that though **41** was less reactive than the 2-*O*-benzyl derivative **42** it was nevertheless more reactive than the conventionally armed donor and could smoothly be coupled on the 4-OH group of the armed thioglycoside **43** without competing self-condensation of **43**.

The Yang group has also investigated superarmed galactothiofuranosides [38]. In line with the findings described above they found that the donor reactivity increased with the number of TBS protective groups in the molecule. However, the 3,5-di-*O*-TBS-2,6-di-*O*-benzoyl derivative was sufficiently reactive to glycosylate partially benzoylated thioglycosides with high chemoselectivity and was therefore used in a range of high yielding oligosaccharide syntheses [38].

**Figure 4:**
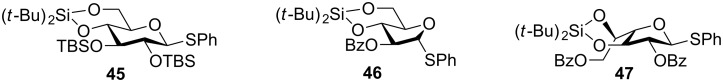
Donors disarmed by the di-*tert*-butylsilylene protective group.

The bifunctional silicon protective group DTBS (Figure 1) has been used both to increase and decrease the reactivity of glycosyl donors. The 4,6-*O*-DTBS-protected thioglucoside **45** was found to be much less reactive than **20** and only couples to armed donor/acceptors in low yield (Figure 4) [24]. This is analogous to the effect of the very similar benzylidene group, which is deactivating the donor partially due to locking the structure in an unreactive conformation and due to the electronic effect of a *trans*-gauche conformation of the hydroxymethyl group [22,39].

Yang and collaborators found that **46** was less reactive than the fully benzoylated analogue, which is obviously also due to the DTBS group locking the molecule into an unreactive conformation [29]. In line with this, the analogue of **46** having a TIPDS group rather than a DTBS was not particularly unreactive, as it is more flexible due to the bigger ring. The Yang group used **46** in a one-pot synthesis of a trisaccharide, where they took advantage of **46** being less reactive than partially benzoylated arabinofuranosides [29]. The concept was extended to the galactofuranosyl series, but was less useful there [38]. A slightly lower reactivity of **47** was found relative to the fully benzoylated species.

**Figure 5:**
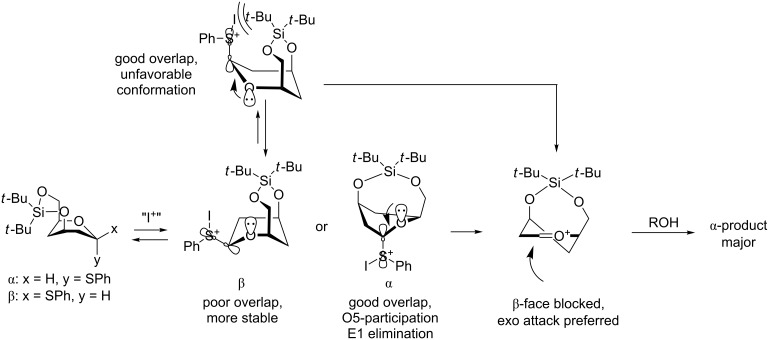
The influence of a 3,6-*O-*tethering on anomeric reactivity and glycosylation selectivity. The α-thioglycoside is more reactive as a conformational change is not needed to expel the sulfonium ion. This is not the case with the β-anomer. Selectivity is mainly controlled by sterics and hence the α-glycoside is kinetic product as the alcohol approach the oxocarbenium ion intermediate from the exo-side.

DTBS groups can also be used to increase the reactivity of glycosyl donors [40]. A series of differently configured monosaccharide thioglycosides were subjected to linking the 3 and 6-OH group together with this silyl ether. This forces the glycosyl-donor conformation to change into an axial-rich conformation and hence into a superarmed donor (Table 2) making it possible to glycosylate an armed glycosyl donor selectively. This approach works for glucosides, mannosides, and galactosides and both, α- and β-thioglycosides [40]. It was shown by competition experiments that these tethered donors were even more reactive than the TBS-protected donors such as **20**. This was particularly the case for the α-anomers as a considerable reactivity difference between α- and β-thioglucosides was observed with the α-anomer consistently being more reactive. This suggested that the exact alignment of the leaving group is important for the reactivity, but a similar difference was not observed for other superarmed glycosyl donors (Figure 5).

**Table 2:** Reactions of 3,6-*O*-silyl-tethered thioglycosides.

Silylated donor	Benzylated donor	Product^a^ (yield %)

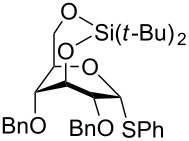 **48**	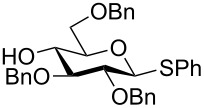 **21**	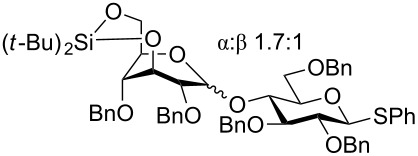 **49** (64%)
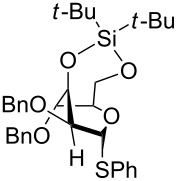 **50**	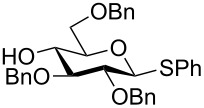 **21**	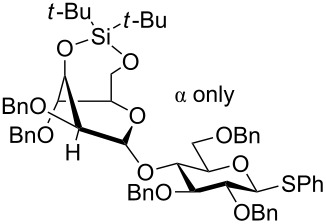 **51** (70%)
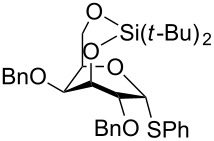 **52**	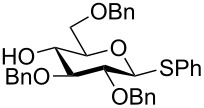 **21**	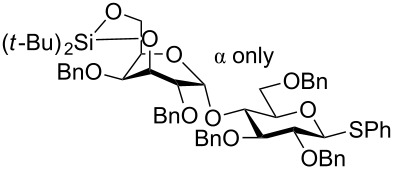 **53** (51%)

^a^Only the shown stereoisomer was obtained. Data taken from [40].

Surprisingly, a 2,4-*O*-tethering of a glucosyl donor, giving the all axial conformation, did not increase the reactivity and the donor was found not to be superarmed. The explanation for this relates to the more strained conformation which counteracts a flattening of the conformation when approaching an sp^2^-hybridized C1 in the TS [41].

### Effect of silyl protective groups on the selectivity

**Scheme 9:**
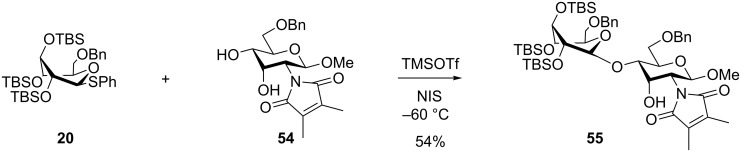
Regio- and stereoselective glycosylation using the superarmed thioglycoside donor **20**.

The bulkiness of TBS groups in donors such as **20** can have a significant influence on the diastereoselectivity. Thus glycosylations with **20** (Table 1) gave exclusively the β-glucoside presumably due to steric hindrance for attack from the α-side [24,42]. The bulkiness of **20** was clearly seen in regioselective glycosylations performed by Felice et al. [43]. So, the glycosylation of the D-*allo*-configured acceptor **54** with **20** not only gave exclusively the β-glucoside, but resulted also in the glycosylation exclusively at the equatorial 4-OH group presumably due to the bulkiness of the silylated donor. Thus compound **55** (Scheme 9) was formed as the only product out of four possible isomers in 54% yield. When the D-*gluco*-configured acceptor analogue of **54** was used, a mixture of regioisomers was obtained.

**Scheme 10:**
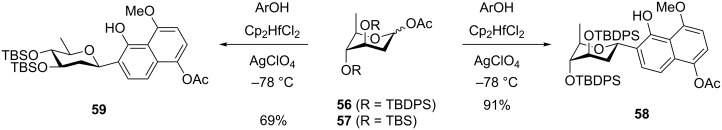
Superarmed donors used for *C*-arylation and the dependence of the size of the silylethers on the stereochemical outcome.

However, the ability of the bulky silyl groups to alter the conformation of the glycosyl-donor ring can be used to control the selectivity. Suzuki and collaborators showed that the *C*-arylation reactions with the 3,4-*O*-di(*tert*-butyldiphenylsilyl)-protected acetate **56** led to the α-glycoside **58** with high selectivity (Scheme 10). The reason for this selectivity is that the equatorial position is more accessible for attack [44]. However, if different protective groups and even the related TBS group were used, predominantly the β-glycoside **59** was obtained in a 14:1 α:β ratio.

A similar conformation-controlled stereoselectivity has been demonstrated in radical reactions, however, with the twist that stereoselectivity here is opposite. The reduction of the selenoglycosides (analogues to **20**) with tributyltin deuteride gave predominantly deuterium in the β-position for silylated derivatives in the ^1^*C*_4_ conformation, because the reaction intermediate is a radical that prefers to be axial. On the other hand, with acetate protective groups, the addition of deuterium occurred predominantly from the β-side [45–46]. The principle of conformational stereocontrol was also used for the stereoselective addition of carbon radicals [46–47].

This selectivity has also been demonstrated for electrophilic additions to the anomeric position. Shuto and collaborators showed that, while 2,3,4-tri-*O*-benzylxylopyranosyl fluoride reacted with allyltrimethylsilane and BF_3_ to give a mixture of α- and β-1-*C*-allyl xylosides, the 2,3,4-*O*-TBS-protected fluoride which is in ^1^*C*_4_ conformation, exclusively gave the β-xyloside. In contrast the xylosyl fluoride with a butane-2,3-bisacetal protective group, that keeps the conformation fixed in a ^4^*C*_1_ conformation, only gave the α-xyloside [48]. This sort of behavior fits well with the reaction model proposed by Woerpel for these types of reactions [49].

**Scheme 11:**
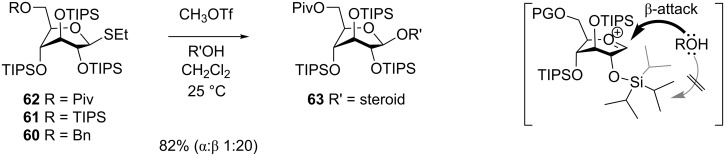
β-Selective glucosylation with TIPS-protected glucosyl donors. The α-face is shielded by the bulky 2-*O*-TIPS protective group.

Yamada and collaborators were the first to show that this principle could be used for the stereoselective synthesis of *O*-glycosides [42]. They prepared thioglucosides **60**–**62** (Scheme 11) having 2,3,4-*O*-TIPS groups and either TIPS, benzyl or pivaloyl protective groups on the 6 position. These glucosyl donors were found to adopt the ^3^*S*_1_ conformation and when they were reacted with methyl triflate and a glycosyl acceptor at room temperature they gave the β-glucosides in 45–92% yield and with 6:1 or better selectivity. The 6-*O* pivaloyl derivative **62** gave the best stereoselectivity (Scheme 11). The technique was later used in the synthesis of the natural product davidiin [50]. The 6-*O*-(3,5-diacetoxy-4-methoxy)benzoyl analogue of **62** was reacted with 3,5-diacetoxy-4-methoxybenzoic acid in the presence of methyl triflate, which gave the β-ester in 83% yield, showing that the principle works for ester synthesis, too.

**Scheme 12:**
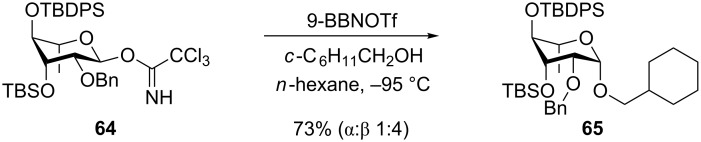
β-Selective rhamnosylation with a conformationally inverted donor.

The Yamada group also attempted to synthetize β-rhamnosides using this principle of conformational inversion [51]. The 3-*O*-TBS-4-*O*-TBDPS-protected trichloroacetimidate **64** was investigated and could give β-selectivity up to 4:1 (Scheme 12). The corresponding thioglycoside donor gave an almost fifty–fifty selectivity. Experiments performed with the 3,4-*O*-TIPS-protected thiorhamnoside donors (Table 1) were not more successful as the activation of this donor with NIS/TfOH also gave mixtures and often predominantly the α-rhamnoside [52]. This, together with the results with the α-selective TBS-protected mannosyl and galactosyl donors (Table 1) [24], shows that there is no general trend with respect to the selectivity of these donors.

On the other hand, the configurationally inverted fully TBS-protected phenyl thiorhamnoside was found to be highly α-selective (Table 1) presumably due to steric hindrance from the 2-*O*-TBS on the β-face. This donor was recently used in the synthesis of glycosyltransferase acceptor substrates [53]. Yet the 2-*O*-TBS protection does not always have this effect. In a recent paper it was shown that in a 4,6-*O*-benzylidene-protected thioglucoside donor, which has been shown by Crich to be α-selective, the α-selectivity increased even more when a 2-*O*-benzyl was exchanged with 2-*O*-TBS or 2-*O*-TIPS [54]. The authors suggested that the silyl group had an inductive effect that favored α-formation.

**Scheme 13:**
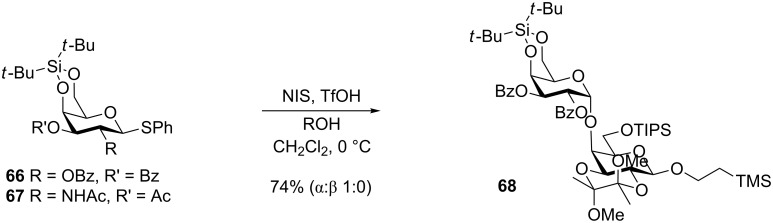
α-Selective galactosylation with DTBS-protected galactosyl donors.

The 4,6-*O*-DTBS group has been shown to be an α-directing group in galactosylation reactions. Kiso and co-workers found that the galactosyl donor **66** (Scheme 13) reacted with several different acceptor alcohols giving exclusively the α-galactoside despite having a potentially β-directing benzoate group in the 2-position [55]. Thus the glycoside **68** was obtained in 74% yield as the only isolated product (Scheme 13). Equally remarkable is that the corresponding DTBS-protected galactosamine donors (such as **67**) displayed the same selectivity in the presence of the silyl group and thereby overriding the influence of a 2-phthalimido, *N*-Troc or *N*-Ac group. It was suggested that the bulky DTBS group is shielding the β-face and thereby preventing attack from that face of the oxocarbenium ion. This methodology has been applied to the synthesis of glycolipids and was shown to also work with 2-*O*-benzyl [56] or 2-*O*-TBS and with *N*-phenyltrifluoroacetimidate as the leaving group [57–58]. A somewhat similar influence has been observed with the much less steric demanding 4,6-*O*-benzylidene protective group [59].

**Scheme 14:**
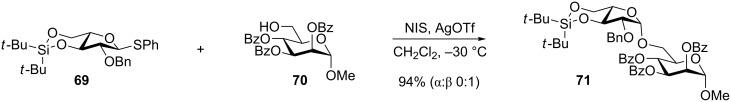
β-Selective arabinofuranosylation with a DTBS-protected donor.

A related stereoselectivity is induced by the DTBS group in arabinofuranosylations. Boons and collaborators found that the 3,5-DTBS-protected L-arabinosyl donor **69** upon reaction with acceptor **70** and activation with NIS/silver triflate gave exclusively the β-glycoside in a yield of 94% (Scheme 14).

Similarly, the reaction of **70** with the corresponding perbenzylated donor only gave a 2:1 β:α-ratio of **71** [60]. It was proposed that the selectivity was caused by a favored β-attack on the oxocarbenium ion in an *E*_3_ conformation as the corresponding α-attack would lead to an unfavorable eclipsed conformation. The exchange of the 2-*O*-benzyl with a 2-*O*-TIPS leads to some erosion of stereoselectivity though the donor was still highly β-selective [61]. Independently of the work by the Boons group, Crich et al. showed that using preactivation conditions on the equivalent D-arabinofuranosyl donor resulted in rupture of the β-selectivity [62]. Ito and co-workers studied the influence of tethering the 3- and 5-OH by a 3,5-*O*-(tetraisopropyldisilylene)acetal and also found the arabinofuranolysations to be β-selective, despite the more flexible system [63]. Interestingly it was recently found that exchanging the 3,5-DTBS group with trifluoroacetates retained a high β-selectivity, which suggests that the stereoselectivity is also related to the deactivating properties of the protective group [64].

**Scheme 15:**
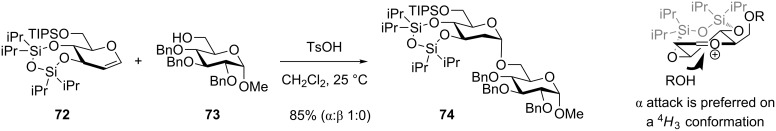
α-Selective glycosylation with a TIPDS-protected glucal donor.

Cyclic silyl protective groups were also recently found to have a beneficial influence on the α-selectivity obtained in glycosylations using glucals [65]. The reaction of 3,4-*O*-TIPDS-protected glucal **72** with acceptor alcohols such as **73**, catalyzed by *p*-TsOH, gave exclusively α-glucoside **74** (Scheme 15). When the same glycosylation was performed with the fully benzylated or TBS-protected glucal the reaction gave a lower yield and was accompanied by some formation of the β-anomer and some Ferrier rearrangement product. With donor **72** the reaction was however high yielding and exclusively α-selective for a range of alcohols. Surprisingly the 6-deoxy version of **72** gave a lower α-selectivity. The observations were explained with the assistance of DFT calculations as being due to the TS structure (formed from **72**) being in an α-selective ^4^*H*_3_ conformation with the 6-TIPS group in an electronically favored gauche–gauche conformation [66], that causes additional shielding from the β-face [65].

**Scheme 16:**
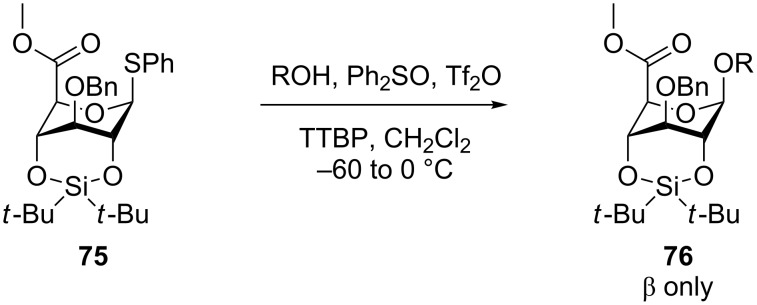
Highly β-selective glucuronylation using a 2,4-DTBS-tethered donor.

The influence of having a 2,4-*O*-di-*tert*-butylsilylene (DTBS) in a glucosyl donor was, as earlier mentioned, not increasing the reactivity of the donor, but it influences the selectivity in the glycosylation. The α-site of the donor becomes the *endo* face, which results in an attack from the β-site. In a conventional glucosyl donor this leads to a 1:10 β-selectivity [41]. Recently this behavior has been used by Furukawa et al. in a β-controlled glucuronylation, where the bulky silylene in **75** ensures high selectivity without neighboring group participation (Scheme 16) [67].

## Conclusion

Much indicates that glycosyl donors with silyl protective groups generally are more reactive than their alkylated counterparts presumably due to the *O*-silyl group being slightly less electron withdrawing than, e.g., a benzyl group. However, the reactivity increase is further augmented when bulky silyl groups, that cause a conformational change to an axial-rich conformation, are present. Such “superarmed” donors have a reactivity beyond what is obtained conventionally because the axial or pseudoaxial OR groups are less electron withdrawing. On the other hand, when the conformation is restricted by cyclic silyl protective groups (i.e., DTBS and TIPDS) and equatorial rich, comparatively unreactive donors result. Similarly, DTBS groups can be used to create superarmed donors by locking the conformation in an axial-rich state.

The silyl groups can also profoundly influence the stereoselectivity but in less obvious ways. Many TBS-protected donors are stereoselective – in some cases selectivities appear to be caused by steric hindrance from the 2-*O*-TBS group. For *C*-glycosides it has been possible to obtain conformationally derived stereocontrol so that persilylated donors adopting a ^1^*C*_4_ conformation give the β-products. However, for *O*-glycosylation, this type of selectivity has been difficult to achieve.

Some very useful stereoselectivities are obtained with DTBS and TIPDS-protected galactosyl, mannosyl and arabinosyl donors. Here the selectivity is very much related to the conformational restriction and face-discrimination imposed by the cyclic silyl group upon the system.
